# The effect of research on the perceived quality of teaching: a cross-sectional study among university students in Lebanon

**DOI:** 10.1186/s12909-023-03998-8

**Published:** 2023-01-17

**Authors:** Hala Sacre, Marwan Akel, Chadia Haddad, Rony M. Zeenny, Aline Hajj, Pascale Salameh

**Affiliations:** 1 INSPECT-LB (Institut National de Santé Publique, d’Épidémiologie Clinique Et de Toxicologie-Liban), Beirut, Lebanon; 2 School of Pharmacy, Lebanese International University, Beirut, Lebanon; 3 School of Education, Lebanese International University, Beirut, Lebanon; 4 School of Medicine, Lebanese American University, Beirut, Lebanon; 5 Research Department, Psychiatric Hospital of the Cross, Jal Eddib, Lebanon; 6 School of Health Sciences, Modern University for Business and Science, Beirut, Lebanon; 7 Department of Pharmacy, American University of Beirut Medical Center, Beirut, Lebanon; 8 Laboratoire de Pharmacologie, Pharmacie Clinique Et Contrôle de Qualité Des Médicament, Faculty of Pharmacy, Saint Joseph University of Beirut, Beirut, Lebanon; 9 Faculté de Pharmacie, Université Laval, Québec, Canada; 10 Oncology Division, CHU de Québec Université Laval Research Center, Québec, Canada; 11 Faculty of Pharmacy, Lebanese University, Hadat, Lebanon; 12 Department of Primary Care and Population Health, University of Nicosia Medical School, 2417 Nicosia, Cyprus

**Keywords:** Research, Education, Teaching evaluation, Quality teaching, Student

## Abstract

**Introduction:**

The complementarity between research and teaching is still debated, although several higher education institutions require instructors to do research. In the absence of a consensus on this matter and given the lack of related studies in Lebanon, this study aimed to describe students’ perception of research integration into teaching, and to link research and professional skills to quality teaching, using validated scales related to these concepts.

**Methods:**

A standardized questionnaire was diffused to university students; it included validated scales: the Student Perception of Research Integration Questionnaire (SPRIQ), the Adapted-Teachers’ quality assessment questionnaire (A-TQAQ), the Student Evaluation of Teaching short form (SET37-QS), and Knowledge and Attitudes Towards Health Research Questionnaire.

**Results:**

Research integration was well perceived, and teaching was well evaluated by 445 participants from various disciplines, particularly those of a higher socioeconomic level, majoring in health, and females, as indicated by their mean scores. Research-active instructors had a significantly better-perceived teaching quality (17%; *p* < 0.001) than their non-active counterparts. This finding was particularly true among postgraduate and higher GPA students. The multivariable analysis showed that the knowledge and attitude towards research were related to students’ better perception of research integration and higher evaluation of teaching.

**Conclusion:**

This study showed an overall good perception of research and teaching evaluation among participants from various disciplines, with research-active instructors having a better-perceived teaching quality. These findings could guide decisions on research integration into curricula using multidisciplinary methodologies to strengthen research integration and involve students in research activities.

**Supplementary Information:**

The online version contains supplementary material available at 10.1186/s12909-023-03998-8.

## Introduction

Academic teachers and researchers are at the front line of teaching scientific literacy to society in areas such as health and the environment. While the purpose of research is to advance knowledge, the main goal of teaching is to build and improve abilities [1], with teaching and research enhancing each other, where active research makes knowledgeable teachers, and teaching inspires creativity and enthusiasm for research. Researchers could convert the methodologies they utilize in their academic activity into an inductive teaching environment by adopting components of their research or selecting challenges more relevant to the themes and levels of the courses taught. As a result, education in the classroom setting would be enhanced by introducing scientific papers and offering students adequate training in the abilities necessary for graduate studies and research careers [1, 2]. More importantly, students would engage in research activities, co-publishing with their teachers, and would develop critical thinking and problem-solving capabilities that would benefit them in any career path they choose [1, 3].

Teachers may play a critical role in delivering scientific literacy to students, with many having access to various sources of knowledge [4]. Some educators might come from a school-teaching background; hence, teaching would be their first-order skill [5]. Their research knowledge is not usually extensive, and for many, it is restricted to methodology classes and research related to their master’s thesis or dissertation [5]. For this group of teachers, research knowledge will be a second-order skill [5]. However, in academia, things are different. Indeed, instructors must continually develop their knowledge, which necessitates a periodic update of scientific knowledge and abilities, to fulfill their teaching role effectively [4, 6].

Since the late 1800s, studies have argued whether teacher and research work duties complement each other or conflict [7]. Faculty research and teaching have long been seen as distinct activities [8–10], as both are full-time careers. Hence, time spent on one activity is usually time taken from the other, particularly when lecturers with heavy research loads, demanding managerial obligations, and deadline schedules for grant and research articles are required to teach as part of their responsibilities [11]. Similarly, instructors with a heavy teaching load might not have time to develop a consequential research path. Ronald Barnett, who perceives that teaching and research are mutually exclusive, claims that faculty members’ preoccupation with research interferes with teaching or that teaching takes up valuable research time [12]. Although it is simpler to manage research and teaching as distinct activities, the research/teaching nexus has been described as symbiotic and synergistic [13,14], while teaching is often presented as a barrier to research excellence [15].

In scientific fields, separating research and teaching contradicts the goal of training the next generation of highly skilled scientists. Indeed, most scientists are passionate about improving education and would welcome opportunities to further combine the two fields [11]. A recent study showed that the quality concerns of teacher-researchers overlap with those of researchers, broadening the meaning of some quality issues, adding new concerns, and omitting others [16].

Several studies examined the research/teaching nexus [17–19] from students’ perspectives, including perception, understanding, experience, and satisfaction, raising debates about the matter and identifying several advantages and barriers. Evidence showed that students valued research [20] and understood the relationship between research and teaching, but this understanding changed across their study cycle with the infusion of research into their learning [21]. Although students believe that the research/teaching nexus help them develop thinking skills, the reported types of skills differed according to their study major [22]. Their satisfaction with research depended mainly on teachers’ epistemiologies and scholarly activities, university prestige, and a supportive learning environment [23]. Nevertheless, students reported several barriers [17,18] and challenges [24–26] to engaging in research during their undergraduate studies, including low exposure to research activities, difficult access to information sources, language barriers (for non-English speakers), insufficient research budgets, inability to write formal research proposals, lack of mentorship and guidance, inadequate time and priorities, and limited understanding of biostatistics.

In Lebanon, despite the presence of many higher education institutions (some of which are research-intensive), no study has ever been conducted to show the potential association between research and teaching quality. As the research-teaching nexus is still debatable and given the lack of related studies in Lebanon, this study aimed to describe students’ perception of research integration into teaching, linking research, academic, and professional skills to quality teaching using validated scales related to these concepts.

## Methods

### Study design and sampling

A cross-sectional online survey of 445 students was performed in Lebanon between March and May 2022 using the non-random snowball sampling technique to collect data from university students. The questionnaire was developed, then created on Google Forms (https://forms.gle/7ea86dWBBuneq8B6A) and distributed on social media (WhatsApp, LinkedIn, and Facebook). The students approached were from the first to final academic years, and postgraduate students. They were enrolled in different faculties of private and public universities. The inclusion criteria were: being a university (undergraduate or graduate) student, age above 18 years, with internet access. Participation in the study was voluntary, and participants received no compensation in exchange for their participation.

It is noteworthy that university students in Lebanon are enrolled in public and private universities [27], although no official figures are available regarding the current distribution of students. In the academic year 2019–2020, students were estimated to be distributed over the Lebanese University (the only public university, tuition-free; around 79,000 students), the Lebanese International University (the largest private university, relatively inexpensive; about 20,000 students), and other private and more expensive universities, including the American University of Beirut (AUB), the Saint Joseph University of Beirut (USJ), the Lebanese American University (LAU), the University of Balamand (UOB), Beirut Arab University (BAU), and the Modern University for Business and Science (MUBS), which have 120,000 students enrolled.

### Questionnaire

The study focused on the perception of students of the teaching and research skills of instructors. The self-report anonymous questionnaire was available in English (Appendix) and consisted of two sections.

The first section included sociodemographic and student characteristics such as age, gender, area of residence, marital status, current academic year, current university, GPA level, the highest level of education, the study major, employment status, and monthly income. The latter was divided into no income, low (< 1.500.000 LL), moderate (1.500.000–3.000.000 LL), and high (> 3.000.000 LL). A question was also added to inquire if the student regularly checks if the instructors carry out research activities or have published articles for adjustment purposes.

The second section included the following assessment scales:

#### The Student Perception of Research Integration Questionnaire (SPRIQ)

This questionnaire was designed to identify how students perceive research integration into their courses [28]. It consists of 40 items graded on a 5-point Likert scale from 1 (very rarely) to 5 (very frequently). The SPRIQ is divided into three constructs, i.e., research integration, quality of the course, and beliefs about research integration [28]. All items were summed, and a total score was created, with a higher score indicating a better perception of research integration.

#### The Adapted-Teachers’ Quality Assessment Questionnaire (A-TQAQ)

The TQAQ was designed to measure the teacher’s academic qualification, professional qualification, and years of experience [29]. In this study, the questionnaire was adapted by including four additional items deemed suitable and necessary to create the A-TQAQ, i.e., “Research activity of an instructor is dependent on one’s academic qualification”, “Excellent mastering of one’s subject as an instructor is dependent on one’s research activity”, “Students taught by more experienced researchers perform academically better”, and “A researcher is a role model for students”. This scale is graded on a 5-point Likert scale ranging from 1 (most unlikely) to 5 (most likely) [29]. All items were summed to yield a total score, with higher scores indicating a better perception of students of their instructors’ qualifications.

#### The Student Evaluation of Teaching—Short Form (SET37-QS)

This tool measures students’ subjective perception of learning and overall satisfaction with a course [30]. It helps obtain student feedback on internal practices and processes that monitor and enhance the quality of higher education instruction [30]. Derived from the SET37 [31], the SET37-QS consists of nine items rated on a 5-point Likert scale from 1 (strongly disagree) to 5 (strongly agree) [30]. The total score was calculated by summing all nine items, with higher scores indicating a better evaluation of teaching quality. In this study, the scale was used twice; in the first instance, students were asked to answer when thinking about the instructor with the highest research activity and then when thinking about the instructor with the lowest research activity in their institution. The variation between the highest and lowest level of the SET37-QS was calculated, and a new variable was created, i.e., the difference between the student evaluation of teaching quality (highest vs. lowest); this variation estimated the difference in perception of teaching quality between instructors according to their research activity (high-caliber researchers versus non-researchers).

#### The knowledge and attitudes towards health research questionnaire

In this scale, ten multiple-choice questions assess knowledge [32], and the proportion of correct answers is determined for each student as a measure of knowledge score [32]; six questions examine their attitudes toward research, with each response being rated on a scale from 0 (unfavorable attitude) to 1 (favorable attitude) [32]. Since the study was intended for all students from health and non-health specialties, the term “health” was removed. These scales were validated in a separate paper and showed to have appropriate structure validity and reliability (submitted article).

### Statistical analysis

Data were analyzed using SPSS software version 25. The principal component analysis was used to assess the construct validity of the used scales, and Cronbach’s alpha was calculated to assess their reliability (internal consistency). A confirmatory factor analysis was carried out to assess the structure of the scales used. Several goodness of fit indicators were re-ported: the Relative Chi-square (χ2/df) that serves as goodness of fit index (cut-off values: < 2–5), the Root Mean Square Error of Approximation (RMSEA) that tests the fit of the model to the covariance matrix (close and acceptable fit are considered for values < 0.05 and < 0.11, respectively), the Goodness of Fit Index (GFI), the Adjusted Goodness of Fit Index (AGFI), comparative fit Index (CFI) and Tucker Lewis Index (TLI) (acceptable values are ≥ 0.90) [33].

A descriptive analysis was done using counts and percentages for categorical variables and means and standard deviations for continuous measures. The sample was normally distributed, as checked by visual inspection of the histogram, and skewness and kurtosis were below |1.96| [34]. In addition, the normality of scales was verified by the normality line of the regression plot and scatter plot of the residual. After checking the normality of both variables (perception of teaching score and assessment of the quality of teaching score), the independent-sample t-test was used to compare the means between two groups, the dependent-sample t-test to compare dependent groups, and the ANOVA test was applied to compare three or more non-dependent means. A *p*-value < 0.05 was considered significant.

All variables that showed a *p*-value < 0.2 in the bivariate analysis were included in all models to avoid potential confounders. A multivariate analysis of covariance (MANCOVA) was carried out, taking the perception of teaching scale and the assessment of the quality of teaching scale as the dependent variables. Moreover, the variable “difference in the evaluation of teaching quality between the highest and the lowest research-active teachers” was considered a dependent variable in the multivariable linear regression analysis.

## Results

### Sample description

Table 1 shows the sociodemographic and other characteristics of the participants. The majority of participants were single (96.0%), female (67.6%), lived in an urban place (75.3%), and 29.9% were employed. Half of the university students were from the LIU (50.1%) and 22% from the LU; most of them were undergraduate students (71.9%) from the first and second academic year (73.1%), and 26.3% had a GPA below 80. More than half of the participants majored in health, medicine, and science (51.9%), and 68.1% regularly checked if their instructors were involved in research activities or had published articles. The mean age of the participants was 20.12 ± 2.79 years.

**Table 1:** Sociodemographic characteristics of the participants (*N* = 445)

Variable	N (%)
**Gender**	
Male	144 (32.4%)
Female	301 (67.6%)
**Area of residence**	
Beirut	234 (52.6%)
Mount Lebanon	98 (22.0%)
North	53 (11.9%)
South	25 (5.6%)
Beqaa	35 (7.9%)
**Place of living**	
Urban	335 (75.3%)
Rural	110 (24.7%)
**Marital status**	
Single/widowed/divorced	427 (96.0%)
Married	18 (4.0%)
**Current academic year**	
First	192 (43.1%)
Second	134 (30.1%)
Third	53 (11.9%)
Four and above	31 (7.0%)
Graduate students	35 (7.9%)
**GPA level**	
≤ 80	117 (26.3%)
81 – 90	67 (15.1%)
> 91	87 (19.6%)
Unknown	174 (39.1%)
**Current University**	
Lebanese University (LU)	98 (22.0%)
Lebanese International University (LIU)	223 (50.1%)
Other universities^*^	124(27.9%)
Highest level of education^**^	
PharmD/MD	17 (3.8%)
Doctorate (PhD, DBA, DPT, etc.)	11 (2.5%)
Master’s degree (MBA, MPH, etc.)	24 (5.4%)
Bachelor’s degree	55 (12.4%)
I am not a graduate student	320 (71.9%)
Others	18 (4.0%)
**Major of study**	
Health, medicine, and science	231 (51.9%)
Other majors	214 (48.1%)
**Employment status**	
Full-time employee	54 (12.1%)
Part-time employee	79 (17.8%)
Unemployed	312 (70.1%)
**Checking if the instructors are involved in any research activity or have any published articles**	
Yes, always	93 (20.9%)
Yes, sometimes	210 (47.2%)
No	142 (31.9%)
	**Mean ± SD**
**Age**	20.12 ± 2.79

^*^Other universities include the American University of Beirut (AUB), the Saint Joseph University of Beirut (USJ), the Lebanese American University (LAU), the University of Balamand (UOB), Beirut Arab University (BAU), and the Modern University for Business and Science (MUBS)

^**^Degree could be earned from a previous educational institution

### Description of the scales used

Table 2 describes the medians, means, SDs, and ranges of the scales used in this study. All means and medians were high, except for research knowledge. There was a moderate but significant difference (17%) between the mean SET37-QS when applied for instructors with the highest versus the lowest research activity (*p* < 0.001).

**Table 2:** Description of the scales used in the study

	**Median**	**Mean**	**Mean %**	**95% CI**	**Min**	**Max**
**Student Perception of Research Integration Questionnaire**	109.00	105.46	73.23	103.04; 107.88	36.00	144.00
Perception of students about integration of research	63.00	61.09	72.72	59.63; 62.55	21.00	84.00
Belief of the student about an educator who is also a researcher	45.00	44.37	73.95	43.31; 45.42	15.00	60.00
**Student Evaluation of Teaching short form (teacher with highest research activity)**	34.00	32.11	71.35	31.24; 32.98	9.00	45.00
**Student Evaluation of Teaching short form (teacher with lowest research activity)**	27.00	27.53	61.17	26.80; 28.26	9.00	45.00
**Student Evaluation of Teaching Short Form difference between highest and lowest research-active instructors**	2.00	4.58	16.63	3.84; 5.31	-20.00	36.00
**Adapted-Teachers’ Quality Assessment Questionnaire**	69.00	68.64	76.26	67.52; 69.76	25.00	90.00
Academic qualifications	34.00	33.47	74.37	32.84; 34.11	9.00	45.00
Professional qualifications	36.00	35.16	78.13	34.58; 35.74	12.00	45.00
**Knowledge about research**	3.00	2.72	30.22	2.56; 2.88	0	9.00
**Attitude towards research**	4.00	4.32	61.71	4.16; 4.48	0	7.00

*CI* Confidence interval for the mean, *Min* Minimum, *Max* Maximum

### Factor analysis

The Promax rotated matrix of the SPRIQ produced two factors with an eigenvalue > 1, accounting for a variance of 63.44% (Bartlett sphericity test *p* < 0.001, KMO = 0.979; Cronbach’s alpha = 0.980) (Supplementary Table S1). The two constructs were termed as follows: Perception of students about research integration and Beliefs of students towards an educator who is also a researcher.

For the A-TQAQ, the explained variance was 57% (Bartlett sphericity test *p* < 0.001, KMO = 0.933; Cronbach’s alpha = 0.937). The factor analysis yielded two factors: Academic Qualifications and Professional Qualifications (Supplementary Table S2).

As for the two SET37-QS scales (highest and lowest research activity), the Promax rotated matrix produced one factor with an eigenvalue > 1, accounting for a variance of 79.79% for instructors with the highest research activity (Bartlett sphericity test *p* < 0.001, KMO = 0.964; Cronbach’s alpha = 0.968) (Supplementary Table S3A) and of 71.48% for the instructors with the lowest research activity (Bartlett sphericity test *p* < 0.001, KMO = 0.953; Cronbach’s alpha = 0.950) (Supplementary Table S3B).

A confirmatory factor analysis of the used scales was run using the structure obtained in the factor analysis. The results were statistically satisfying, as displayed by the goodness of fit values (Table S4).

### Bivariate analysis

Table 3 shows the bivariate analysis taking research integration and the evaluation of teaching scales as the dependent variables. The results showed that, compared to males, females had a better perception of research integration and better evaluation of teaching quality. The mean difference between student evaluations was also higher among females. Higher means of research integration perception, higher student evaluation of teaching quality (highest research activity), and higher quality assessment scales were found among those with a higher monthly income compared to the other groups. Higher means of research integration perception and higher student evaluation of teaching quality were found among students from LIU compared to those from other universities. Moreover, significantly higher means of research integration perception and evaluation of teaching quality were found among students who majored in health, medicine, and science.

**Table 3:** Bivariate analysis taking the perception of research integration and the evaluation of teaching scales as the dependent variables

	**SPRIQ**	**SET37-QS (Highest research activity)**	**SET37-QS (Lowest research activity)**	**A-TQAQ (Academic qualification)**	**A-TQAQ (Professional Qualifications)**	**Difference between SET37-QS (High vs. Low)**
	**Mean (95% CI)**	**Mean (95% CI)**	**Mean (95% CI)**	**Mean (95% CI)**	**Mean (95% CI)**	**Mean (95% CI)**
**Gender**						
Male	99.17 (94.65; 103.69)	29.77 (28.14; 31.40)	26.31 (24.96; 27.67)	33.24 (32.04; 34.44)	34.57 (33.50; 35.64)	3.45 (2.13; 4.77)
Female	108.47 (105.66; 111.28)	33.23 (32.23; 34.23)	28.11 (27.24; 28.98)	33.59 (32.84; 34.33)	35.44 (34.76; 36.13)	5.11 (4.22; 6.00)
*p-value*	**0.001**	** < 0.001**	**0.024**	0.613	0.166	**0.039**
**Marital status**						
Single/Widowed/ Divorced	105.54 (103.07; 108.00)	32.23 (31.35; 33.11)	27.52 (26.77; 28.27)	33.49 (32.85; 34.14)	35.18 (34.59; 35.78)	4.71 (3.95; 5.46)
Married	103.66 (88.96; 118.36)	29.22 (24.01; 34.43)	27.77 (23.91; 31.63)	33.00 (29.92; 36.07)	34.66 (32.10; 37.22)	1.44 (-2.21; 5.09)
*p-value*	0.765	0.179	0.894	0.760	0.728	0.086
**Monthly income**						
No income	103.57 (100.71; 106.43)	31.87 (30.87; 32.88)	27.37 (26.47; 28.28)	32.20 (31.39; 33.00)	34.27 (33.56; 34.98)	4.49 (3.57; 5.42)
Low (< 1.500.000 LL)	98.75 (91.64; 105.85)	30.05 (27.41; 32.70)	26.20 (24.05; 28.36)	34.83 (33.39; 36.27)	35.42 (33.77; 37.07)	3.85 (2.07; 5.63)
Moderate (1.500.000–3.000.000 LL)	112.96 (106.40; 119.53)	34.92 (32.51; 37.32)	28.79 (26.92; 30.65)	35.93 (34.27; 37.59)	37.69 (36.24; 39.13)	6.12 (3.67; 8.58)
High (> 3.000.000 LL)	116.32 (107.89; 124.75)	32.76 (29.52; 35.99)	28.71 (26.08; 31.35)	35.63 (33.52; 37.73)	36.58 (34.67; 38.50)	4.04 (1.86; 6.22)
*p-value*	** < 0.001**	**0.024**	0.199	** < 0.001**	** < 0.001**	0.365
**Current university**						
Lebanese University (LU)	99.25 (93.38; 105.12)	29.22 (27.29; 31.15)	25.98 (24.42; 27.55)	33.13 (31.77; 34.48)	34.63 (33.41; 35.85)	3.23 (1.64; 4.82)
Lebanese International University (LIU)	110.46 (107.41; 113.52)	33.83 (32.74; 34.93)	29.10 (28.11; 30.10)	33.52 (32.65; 34.39)	35.06 (34.25; 35.87)	4.73 (3.74; 5.71)
Others	101.37 (96.66; 106.09)	31.29 (29.49; 33.09)	25.92 (24.50; 27.35)	33.66 (32.39; 34.94)	35.77 (34.63; 36.91)	5.37 (3.84; 6.89)
*p-value*	** < 0.001**	** < 0.001**	** < 0.001**	0.834	0.373	0.126
**Current academic year**						
First	104.44 (100.91; 107.96)	32.04 (30.79; 33.29)	27.92 (26.84; 29.00)	32.67 (31.80; 33.53)	34.36 (33.49; 35.23)	4.11 (3.02; 5.21)
Second	104.67 (100.35; 109.00)	31.62 (30.04; 33.20)	27.41 (26.11; 28.72)	33.99 (32.79; 35.18)	35.72 (34.68; 36.76)	4.20 (2.90; 5.51)
Third	107.15 (98.98; 115.31)	32.52 (29.56; 35.48)	25.88 (23.81; 27.95)	33.88 (31.76; 36.00)	35.41 (33.72; 37.10)	6.64 (4.12; 9.16)
Four and above	112.29 (102.28; 122.29)	33.80 (30.06; 37.55)	27.38 (23.65; 31.11)	33.90 (30.68; 37.11)	37.00 (34.57; 39.42)	6.41 (3.06; 9.77)
Graduate students	105.48 (95.62; 115.34)	32.25 (28.94; 35.56)	28.48 (25.51; 31.45)	34.94 (32.93; 36.94)	35.42 (33.15; 37.69)	3.77 (1.45; 6.09)
*p-value*	0.597	0.825	0.505	0.246	0.128	0.160
**GPA level**						
≤ 80	101.35 (96.52; 106.17)	30.87 (29.27; 32.47)	26.57 (25.12; 28.02)	33.07 (31.99; 34.16)	34.87 (33.83; 35.90)	4.29 (2.98; 5.61)
81 – 90	108.61 (101.82; 115.40)	33.26 (30.98; 35.55)	29.02 (27.24; 30.81)	33.32 (31.35; 35.30)	34.77 (33.14; 36.40)	4.23 (2.40; 6.07)
> 91	108.21 (102.73; 113.69)	32.79 (30.84; 34.74)	28.26 (26.57; 29.95)	32.33 (30.68; 33.97)	34.29 (32.84; 35.75)	4.52 (2.63; 6.42)
*p-value*	0.098	0.152	0.089	0.648	0.802	0.969
**Major of study**						
Health, medicine, and science	110.61 (107.60; 113.61)	33.95 (32.88; 35.02)	28.24 (27.31; 29.17)	33.85 (33.01; 34.69)	35.94 (35.17; 36.70)	5.71 (4.67; 6.74)
Other majors	99.91 (96.17; 103.64)	30.13 (28.78; 31.47)	26.77 (25.62; 27.91)	33.07 (32.11; 34.02)	34.32 (33.46; 35.19)	3.35 (2.32; 4.39)
*p-value*	** < 0.001**	** < 0.001**	0.050	0.221	**0.006**	**0.002**
**Employment status**						
Employed	107.46 (102.50; 112.43)	31.54 (29.72; 33.37)	26.95 (25.56; 28.34)	35.52 (34.46; 36.58)	36.29 (35.28; 37.30)	4.59 (3.14; 6.04)
Unemployed	104.61 (101.86; 107.35)	32.35 (31.38; 33.32)	27.78 (26.91; 28.64)	32.60 (31.84; 33.36)	34.68 (33.98; 35.38)	4.57 (3.71; 5.43)
*p-value*	0.321	0.441	0.311	** < 0.001**	**0.012**	0.980
**Checking if the instructors are involved in any research activity or have any published articles**						
Yes	104.80 (101.82; 107.78)	31.89 (30.83; 32.95)	27.84 (26.92; 28.75)	33.37 (32.59; 34.15)	35.11 (34.39; 35.82)	4.05 (3.19; 4.91)
No	106.87 (102.69; 111.05)	32.57 (31.05; 34.09)	26.87 (25.65; 28.09)	33.70 (32.61; 34.79)	35.28 (34.30; 36.26)	5.70 (4.30; 7.10)
*p-value*	0.435	0.473	0.225	0.631	0.789	**0.049**

*SPRIQ* Student Perception of Research Integration Questionnaire

*SET37-QS* Student Evaluation of Teaching short form

*A-TQAQ* Adapted-Teachers’ Quality Assessment Questionnaire

*LIU* Lebanese International University

*LU* Lebanese University

*CI* Confidence interval

^*^Values marked in bold are significant

Also, a higher mean difference between student evaluations was found in graduates and those who majored in health, medicine, and science. Those who were employed had higher means of quality assessment scales compared to non-employed participants. Finally, a positive correlation was found between knowledge about research and research integration perception and evaluation of teaching quality scales. A significantly higher mean attitude about research was associated with a higher research integration perception and higher student evaluation of teaching quality scales (Table 4).

**Table 4:** Correlations analysis between the perception of research integration and the evaluation of teaching scales with age and knowledge and attitude scales

	**SPRIQ**	**SET37-QS (Highest research activity)**	**SET37-QS (Lowest research activity)**	**A-TQAQ (Academic qualification)**	**A-TQAQ (Professional Qualifications)**	**Difference between SET37-QS (High vs. Low)**
	**Correlation coefficient**	**Correlation coefficient**	**Correlation coefficient**	**Correlation coefficient**	**Correlation coefficient**	**Correlation coefficient**
**Age**	0.04	-0.02	0.02	0.09	0.06	-0.04
*p-value*	0.41	0.76	0.70	**0.04**	0.22	0.45
**Knowledge about research**	0.35	0.33	0.15	0.11	0.20	0.24
*p-value*	** < 0.001**	** < 0.001**	**0.002**	**0.02**	** < 0.001**	** < 0.001**
**Attitude towards research**	0.14	0.17	0.14	-0.07	-0.05	0.06
*p-value*	**0.004**	** < 0.001**	**0.003**	0.16	0.25	0.18

*SPRIQ* Student Perception of Research Integration Questionnaire

*SET37-QS* Student Evaluation of Teaching short form

*A-TQAQ* Adapted-Teachers’ Quality Assessment Questionnaire

^*^Values marked in bold are significant

### Multivariable analysis: correlates of research integration, education quality and qualifications scales

The MANCOVA analysis was performed by taking the research perception and teaching quality assessment scales as the dependent variables (Table 5).

**Table 5:** Multivariable analysis using the GLM method

	**Beta**	***P*-value**	**Confidence interval**	
			**Lower**	**Upper**
**Perception of Research Integration Questionnaire**				
Age	-0.52	0.45	-1.89	0.85
Knowledge about research	4.30	** < 0.001**	2.55	6.05
Attitude towards research	2.44	**0.01**	0.71	4.18
Gender (Female vs. Male^*^)	3.28	0.33	-3.30	9.85
Academic year (Graduate students vs. first^*^)	5.38	0.44	-8.38	19.14
Academic year (Four and above vs. first^*^)	13.08	0.07	-1.05	27.20
Academic year (Third vs. first^*^)	7.19	0.16	-2.96	17.33
Academic year (Second vs. first^*^)	4.50	0.22	-2.66	11.66
GPA (Highest vs. lowest^*^)	3.04	0.40	-4.10	10.19
GPA (Average vs. lowest^*^)	4.92	0.20	-2.66	12.50
Monthly income (Highest vs. no income^*^)	13.37	**0.02**	2.55	24.19
Monthly income (Moderate vs. no income^*^)	8.26	0.12	-2.12	18.63
Monthly income (Low vs. no income^*^)	-3.81	0.46	-13.94	6.33
Highest level of education (Graduate vs. undergraduate^*^)	-7.09	0.08	-15.03	0.85
Major of study (Health, medicine, and science vs. other)	6.13	0.05	-0.03	12.28
Current university (LIU vs. other)	8.44	**0.03**	1.04	15.83
Current university (LU vs. other)	-0.91	0.82	-8.96	7.14
Employment status (Employed vs. unemployed^*^)	0.95	0.83	-7.93	9.83
**SET37-QS (highest research activity)**				
Age	-0.35	0.14	-0.83	0.12
Knowledge about research	1.26	** < 0.001**	0.65	1.86
Attitude towards research	0.99	** < 0.001**	0.39	1.59
Gender (Female vs. Male^*^)	1.46	0.21	-0.81	3.72
Academic year (Graduate students vs. first^*^)	2.57	0.29	-2.17	7.32
Academic year (Four and above vs. first^*^)	4.18	0.09	-0.69	9.05
Academic year (Third vs. first^*^)	2.91	0.10	-0.59	6.41
Academic year (Second vs. first^*^)	0.94	0.46	-1.53	3.40
GPA (Highest vs. lowest^*^)	0.82	0.51	-1.65	3.28
GPA (Average vs. lowest^*^)	1.79	0.18	-0.82	4.40
Monthly income (highest vs. no income^*^)	1.53	0.42	-2.20	5.26
Monthly income (Moderate vs. no income^*^)	3.79	**0.04**	0.21	7.37
Monthly income (Low vs. no income^*^)	-0.60	0.74	-4.09	2.90
Highest level of education (Graduate vs. undergraduate^*^)	-1.39	0.32	-4.13	1.35
Major of study (Health, medicine, and science vs. other)	1.76	0.10	-0.37	3.88
Current university (LIU vs. other)	2.34	0.07	-0.21	4.89
Current university (LU vs. other)	-2.16	0.13	-4.94	0.61
Employment status (Employed vs. unemployed^*^)	-1.37	0.38	-4.43	1.69
**SET37-QS (Lowest research activity)**				
Age	-0.03	0.90	-0.46	0.40
Knowledge about research	0.53	0.06	-0.02	1.09
Attitude towards research	0.81	** < 0.001**	0.26	1.35
Gender (Female vs. Male^*^)	0.53	0.61	-1.54	2.60
Academic year (Graduate students vs. first^*^)	0.19	0.93	-4.14	4.52
Academic year (Four and above vs. first^*^)	1.37	0.55	-3.08	5.82
Academic year (Third vs. first^*^)	-0.65	0.69	-3.85	2.54
Academic year (Second vs. first^*^)	0.71	0.54	-1.55	2.96
GPA (Highest vs. lowest^*^)	1.05	0.36	-1.20	3.30
GPA (Average vs. lowest^*^)	2.64	**0.03**	0.25	5.02
Monthly income (highest vs. no income^*^)	3.41	0.05	0.00	6.81
Monthly income (Moderate vs. no income^*^)	3.54	**0.03**	0.27	6.80
Monthly income (Low vs. no income^*^)	0.57	0.73	-2.62	3.76
Highest level of education (Graduate vs undergraduate^*^)	0.67	0.60	-1.83	3.17
Major of study (Health, medicine, and science vs. other)	0.04	0.97	-1.90	1.98
Current university (LIU vs. other)	2.79	**0.02**	0.46	5.11
Current university (LU vs. other)	-0.57	0.66	-3.10	1.97
Employment status (Employed vs unemployed^*^)	-3.87	**0.01**	-6.66	-1.07
**A-TQAQ (Academic qualification)**				
Age	0.08	0.68	-0.32	0.49
Knowledge about research	0.23	0.39	-0.29	0.74
Attitude towards research	-0.43	0.10	-0.95	0.08
Gender (Female vs. Male^*^)	1.08	0.27	-0.86	3.02
Academic year (Graduate students vs. first^*^)	1.34	0.52	-2.71	5.39
Academic year (Four and above vs. first^*^)	0.46	0.83	-3.70	4.62
Academic year (Third vs. first^*^)	0.29	0.85	-2.70	3.27
Academic year (Second vs. first^*^)	0.62	0.56	-1.48	2.73
GPA (Highest vs. lowest^*^)	-0.49	0.65	-2.59	1.62
GPA (Average vs. lowest^*^)	0.35	0.76	-1.88	2.59
Monthly income (highest vs. no income^*^)	2.77	0.09	-0.41	5.96
Monthly income (Moderate vs. no income^*^)	2.44	0.12	-0.61	5.50
Monthly income (Low vs. no income^*^)	2.82	0.06	-0.16	5.81
Highest level of education (Graduate vs. undergraduate^*^)	-1.34	0.26	-3.68	1.00
Major of study (Health, medicine, and science vs. other)	1.32	0.15	-0.49	3.13
Current university (LIU vs. other)	-0.87	0.43	-3.05	1.31
Current university (LU vs. other)	-1.39	0.25	-3.76	0.99
Employment status (Employed vs. unemployed^*^)	1.47	0.27	-1.14	4.09
**A-TQAQ (Professional Qualifications)**				
Age	-0.10	0.58	-0.45	0.25
Knowledge about research	0.53	**0.02**	0.08	0.98
Attitude towards research	-0.38	0.10	-0.82	0.07
Gender (Female vs. Male^*^)	1.58	0.07	-0.11	3.27
Academic year (Graduate students vs. first^*^)	0.33	0.86	-3.21	3.87
Academic year (Four and above vs. first^*^)	1.70	0.36	-1.93	5.33
Academic year (Third vs. first^*^)	0.39	0.77	-2.21	3.00
Academic year (Second vs. first^*^)	1.34	0.15	-0.50	3.18
GPA (Highest vs. lowest^*^)	-0.60	0.52	-2.43	1.24
GPA (Average vs. lowest^*^)	-0.31	0.75	-2.26	1.64
Monthly income (highest vs. no income^*^)	2.26	0.11	-0.52	5.04
Monthly income (Moderate vs. no income^*^)	3.17	**0.02**	0.50	5.84
Monthly income (Low vs. no income^*^)	1.93	0.15	-0.67	4.54
Highest level of education (Graduate vs. undergraduate^*^)	0.06	0.96	-1.99	2.10
Major of study (Health, medicine, and science vs. other)	1.41	0.08	-0.17	3.00
Current university (LIU vs. other)	-1.64	0.09	-3.55	0.26
Current university (LU vs. other)	-1.95	0.06	-4.02	0.12
Employment status (Employed vs. unemployed^*^)	0.52	0.65	-1.76	2.80

^*^Reference group

When considering the perception of research integration questionnaire as the dependent variable, the results showed that higher knowledge (Beta = 4.30) and attitude (Beta = 2.44) about research were significantly associated with a higher perception of research integration in teaching. Also, having a high monthly income vs. no income (Beta = 13.37) and studying at LIU (Beta = 8.43) were significantly associated with a higher perception of research.

Taking the SET37-QS about the highest research activity instructor scale as the dependent variable, the results showed that higher knowledge (Beta = 1.25) and attitude scores (Beta = 0.98) were significantly associated with higher SET37-QS scores. A moderate monthly income (Beta = 3.79) vs. no income was significantly associated with a higher SET37-QS score about highest research activity teacher.

When considering the SET37-QS about the lowest research activity instructor scale as the dependent variable, the results showed that a higher attitude score (Beta = 0.80), an average vs. low GPA (Beta = 2.63), a moderate monthly income (Beta = 3.53) vs. no income, and studying at LIU (Beta = 2.78) were significantly associated with a higher SET37-QS score. However, being employed (Beta = -3.86) was significantly associated with a lower SET37-QS about the lowest research activity instructor score.

Taking the quality assessment questionnaire-professional qualifications as the dependent variable, the results showed that a moderate monthly income (Beta = 3.16) and better knowledge about research (Beta = 0.52) were significantly associated with a higher quality assessment. No significant association was found between all the variables used and the quality assessment questionnaire—academic qualifications (*p* > 0.05 for all).

### Multivariable analysis: correlates of the difference in student evaluations between instructors with highest versus lowest research activity

A linear regression model was performed, taking the difference between student evaluations of teaching quality between the highest and lowest research active instructors as the dependent variable. The results showed that being in the third academic year (Beta = 2.96), being a graduate student (Beta = 3.21), and having better knowledge about research (Beta = 0.88) were significantly associated with a higher difference between student evaluations of teaching quality scales. However, being married (Beta = -4.27), being an undergraduate student (Beta = -3.00), and regularly checking if the instructor is involved in research activities were significantly associated with a lower difference in student evaluations of teaching quality scales (Table 6).

**Table 6:** Multivariable linear regression taking the difference between the student evaluation of teaching short form (highest vs lowest) as the dependent variable

	**Unstandardized Beta**	**Standardized Beta**	**p-value**	**Confidence interval**	
				**Lower Bound**	**Upper Bound**
Gender (Female vs. Male^*^)	0.74	0.04	0.34	-0.79	2.26
Marital status (Married vs. single^*^)	-4.28	-0.11	**0.03**	-8.08	-0.47
Highest level of education (Undergraduate vs. graduate^*^)	-3.00	-0.17	** < 0.001**	-4.89	-1.12
Major of the study (Health, medicine and science vs. other^*^)	1.23	0.08	0.10	-0.24	2.70
Checking instructor if they are conducting any research activities (Yes vs. No^*^)	-1.61	-0.10	**0.04**	-3.12	-0.11
Academic year (Second vs first^*^)	0.36	0.02	0.67	-1.30	2.02
Academic year (Third vs. first^*^)	2.96	0.12	**0.01**	0.68	5.25
Academic year (Four and above vs. first^*^)	3.96	0.13	0.01	1.04	6.89
Academic year (Graduate students vs. first^*^)	3.22	0.11	**0.04**	0.07	6.37
Knowledge about research	0.89	0.19	** < 0.001**	0.45	1.32

Variables entered: gender, marital status, the highest level of education, major of the study, checking if the instructor is involved in any research activity, academic year, and knowledge about research

^*^Reference group

## Discussion

This study aimed to assess university students’ perceptions of research integration into teaching, linking research, academic, and professional skills to quality teaching. Firstly, the scales used in this study have high internal consistency and high values of loading on factors, thus producing valid measures for evaluating the research and quality of teaching since the results have demonstrated excellent reliability and factorial validity, through both exploratory and confirmatory analyses. These well-designed measures, used for assessing student perceptions of research integration into a course and the quality of instruction, have in common that they cover different teaching domains, are based on an educational theory and have been thoroughly examined for validity and reliability at several levels. The differences we found in the content and structure of the various used instruments might be attributed to the expectations and demands of the different institutions [35]; furthermore, these variances might be the result of differences in the quantity and types of participants, as well as methodological approaches.

Overall, research activity and teaching quality were significantly associated, and research-active instructors were perceived significantly better, as measured by the difference in evaluations between instructors. This value permitted to overcome interindividual and interinstitutional differences, thus confirming the association between research and teaching. Indeed, the analysis [15] of more than 2000 documents could demonstrate that research is perceived as a positive element in the context of teaching. Nevertheless, a previous study [36] among a cross-disciplinary sample of academics within a research-oriented university showed a positive relationship between teaching quality and research quality but not research productivity. Further studies are necessary to address this particular aspect of the research-teaching nexus.

Regarding the difference between student evaluations of teaching quality when thinking about the instructor with the highest compared to lowest research activity, our results have shown that instructors with the highest research activities had a better-perceived teaching quality than those with the lowest. This advantage of research-active instructors was significantly more visible in postgraduate students and those with better knowledge about research. Our results are similar to previous findings, showing that master’s students taught by teachers with high-quality publications had higher grades [37]. A possible explanation could be that graduate students and those with more knowledge about research might interpret differently and perceive better the roles of research and teaching in their careers than undergraduate students. Indeed, over an academic year, graduate student participants improved in most teaching and research skills [37].

The results showed an overall acceptable perception of research integration in teaching and teaching evaluation among participants from various disciplines, as indicated by their mean scores and correlations. Our findings are consistent with those of other studies [8, 38–40] that have highlighted the effectiveness of a research-based system as a learning strategy for students in many disciplines. In this study, students were aware of the importance of research throughout their curriculum and perceived well the advantages of integrating research into their courses, likely because several institutions in the country have improved their programs, adding the research experience to their curricula, and students tended to believe that research is vital for their future careers. This finding was visible among female and higher-income students. Students with a high monthly income have a better perception of research integration and a good evaluation of the quality of teaching. Income might affect how people perceive situations. Students with a low income might have inequitable access to effective teachers [41]. Studies have shown that low income might negatively affect student behavior, achievement, and school retention [42,43]. Regarding gender, our results contradict those of previous studies [44,45], showing no association between gender and research perception. This perspective is expected to be in favor of a more balanced gender distribution among future researchers.

Moreover, students’ overall evaluations of their instructors’ teaching skills were good in this study, confirming the appropriateness of Lebanese higher education teaching, although discrepancies might exist between universities and instructors. In higher education across the world, student assessment of teaching is commonly utilized to measure the efficacy of instruction through questionnaires [46]. Indeed, students are seen as customers seeking quality teaching and are the best placed to evaluate teacher performances [47]. Furthermore, quality processes in higher education evaluate teachers systematically to identify strengths and weaknesses, which helps them improve their teaching skills [48], demonstrating the commitment of institutions to monitoring teaching performance as part of ongoing program improvement efforts [48].

The multivariable analysis showed that the knowledge and attitude towards research in general were related to students’ better perception of research integration and higher evaluation of teaching, added to a higher GPA and graduate studies involvement. Other studies among students revealed high knowledge, perception, and positive attitudes towards research [14, 49–51]. Similarly, a Lebanese study among 523 students found a favorable perspective, attitude, and practice toward medical research [52]. In fact, the concepts of knowledge, attitude and perception are complexly correlated: the literature has established that knowledge and perception are closely linked [53]. Knowledge is necessary to comprehend fundamental concepts in the literature, improve broad thinking and communication abilities, and develop professional competency in specializations in the future [54,55]. In addition, attitude is a characteristic of perception; how things are seen and interpreted determines how the person will react or behave [56]. Also, being involved in graduate studies is, by itself, linked to a higher GPA and is associated with a higher exposure to research and researchers. Consequently, further studies are necessary to elucidate many aspects of the complex association between research-related factors and student evaluation of teaching. Research assessing teachers’ perspectives is also warranted to compare attitudes between teachers and students, thus unveiling an essential part related to this topic.

## Limitations

The cross-sectional design of this study does not allow for inferring causality between the student perception of research, evaluation of the quality of teaching, and the associated variables. The sample size might seem insufficient to generalize to the entire university student population (less than 1% of Lebanese university students); however, it was powerful enough to do the intended statistical calculations. In this study, it was not possible to present a flowchart of the participants since the snowball technique was used for data collection. This online technique was used as the university courses were still offered online during the data collection period due to COVID-19 and the severe economic crisis in Lebanon, precluding face-to-face interviews. Thus, the sample might not represent the student population in Lebanon. Since the sample included more students from the LIU than other universities, and more than half of them were health/science students, who were more willing to complete the lengthy questionnaire about science and teaching, the possibility of a selection bias could be increased but would not affect the results of multivariable analyses. Students were selected from different majors; nevertheless, half of the students were from health, medicine, and science majors (51.9%), and research activities could be more predominant in health majors, leading to information bias. There were small effect size differences with the non-health category for the studied scales (although statistically significant). Also, cross-tabulation was done between health/science vs. other majors related to whether they checked their instructors’ research activities; the results (not shown) were not significant, and no difference was found between the two groups. This fact might attenuate the selection bias effect on our results. Another noteworthy point is the use of the English language among university students: although Arabic is still the official language in Lebanon, English is commonly used by youth, mainly due to globalization and social media literacy, as shown in studies [57, 58], thus an effect of this factor is not expected on our results.

This study was carried out during the first semester of the academic year when students might not have enough information and perception about research or their instructor, and data were collected through a self-report questionnaire using a non-random snowball technique, which might have led to information bias. Student perception and teaching evaluation might be biased since it was subjective and not verified in practice. However, assessing subjective perception is required when conducting evaluation surveys related to a service provided to customers (who are the students here). Finally, this study could not explore all related factors; thus, residual confounding bias is possible. Further studies that take into account these weaknesses are necessary to confirm our results.

## Conclusion

This study showed an overall good perception of research and teaching evaluation among participants from various disciplines, with research-active instructors having a better-perceived teaching quality. Students were aware of the importance of research throughout their curriculum and integrating it into their courses and tended to believe that it is critical to their future careers. These findings could guide decisions on research integration into curricula using multidisciplinary methodologies to strengthen research integration and involve students in research activities.

## Supplementary Information

**Additional file 1.**

**Additional file 2:** **TableS1.** Factor analysis of the Student Perception of Research IntegrationQuestionnaire (SPRIQ). ** Table S2.** Factor analysis of the Adapted-Teachers’Quality Assessment Questionnaire (A-TQAQ) Questionnaire.** Table S3. **Factoranalysis of the Student Evaluation of Teaching-short form (SET37-QS). **Table S4.** Confirmatory factor analysis.

## Data Availability

The datasets generated during and/or analyzed during the current study are available from the corresponding author on reasonable request.

## Abbreviations

SPRIQ: Student Perception of Research Integration Questionnaire
A-TQAQ: Adapted-Teachers’ quality assessment questionnaire
SET37-QS: The Student Evaluation of Teaching short form
GPA: Grade point average
LIU: Lebanese International University
AUB: American University of Beirut
USJ: Saint Joseph University of Beirut
LAU: Lebanese American University
UOB: University of Balamand
BAU: Beirut Arab University
MUBS: Modern University for Business and Science
MANCOVA: Multivariate analysis of covariance
SPSS: Statistical Package for the Social Sciences
KMO: Kaiser–Meyer–Olkin
SD: Standard deviation
