# Supplementary material for: The effect of research on the perceived quality of teaching: a cross-sectional study among university students in Lebanon
Source: BMC Med Educ. 2023 Jan 17;23:31. doi: 10.1186/s12909-023-03998-8 (PMC9843099; doi:10.1186/s12909-023-03998-8)
Supplement: Supplementary file 1 — Additional file 1. [file 12909_2023_3998_MOESM1_ESM.docx]

**Role of Research in Improving Teaching Skills**

Dear university student,

You are invited to participate in a study the role of research in improving teaching skills.

This survey is conducted by a group of academic researchers and requires about about 15 minutes to complete.

All the information gathered is anonymous and will be treated confidentially.

Your participation in this study is voluntary.

Thank you in advance for your cooperation,

The research team members.

**Please check all the below statements to proceed to the survey.**

I have read and understood the above information

I understand that my participation is voluntary

I understand that my data will be kept confidential

I agree to participate in this study

**Sociodemographic Characteristics**

1. **Age (in years):**
2. **Gender:** Male Female
3. **Marital status:** Single Married Widowed Divorced
4. **Individual monthly income**

No income <1,500,000 LBP 1,500,000-3,000,000 LBP >3,000,000 LBP

1. **In which region do you live?**

Beirut Mount Lebanon North South Beqaa

1. **Region of living:** Urban Rural
2. **Current university:**

LU  USJ  AUB  LAU  UOB  LIU  BAU  MUBS Other

1. **Current academic year**

First Second Third Fourth Fifth 6^th^-7^th^ (medicine only) Graduate student (Master’s or equivalent, PhD candidate, resident...)

1. **Indicate your overall cumulative GPA (Credits system. Please specify if /4 or /5) or your annual average (French system. Please specify if /20 or /100)**
2. **If you are a graduate student, what is the highest level of education already completed**

PharmD/MD

Doctorate (PhD, DBA, DPT, etc.)

Master’s degree (MBA, MPH, etc.)

Bachelor’s degree

I am not a graduate student

Others: _____________________________

1. **Major of study**

Business Law Agricultural and food sciences Arts and sciences

Education Engineering Health and medicine Sciences Other:

1. **Employment status**

Full-time employee Part-time employee I do not work

1. **Do you generally check if your instructors are conducting any research activities or have any published articles?**

Yes, always  Yes, sometimes No, never

**Research and Teaching Scales**

| **Student Perception of Research Integration Questionnaire (SPRIQ) [1]** | | | | | | | | | | |
| --- | --- | --- | --- | --- | --- | --- | --- | --- | --- | --- |
| **Items** | **Very Rarely** | | **Rarely** | | **Occasionally** | | **Frequently** | | **Very Frequently** | |
| **Do you believe that an educator who is also a researcher would better do the following?** | | | | | | | | | | |
| 1. Integrate knowledge about research findings |  | |  | |  | |  | |  | |
| 1. Teach the students to pay attention to recent developments in the field |  | |  | |  | |  | |  | |
| 1. Give information about studies that have been carried out in my field |  | |  | |  | |  | |  | |
| 1. Teach the students to pay attention to the way research is carried out |  | |  | |  | |  | |  | |
| 1. Inspire the students to learn more about a given discipline |  | |  | |  | |  | |  | |
| 1. Teach the students to pay attention to research methodology |  | |  | |  | |  | |  | |
| 1. Familiarize the students with the research carried out by the instructors |  | |  | |  | |  | |  | |
| 1. Encourage the students not to be satisfied with an explanation too quickly |  | |  | |  | |  | |  | |
| 1. Encourage the students to search for answers to unanswered research questions |  | |  | |  | |  | |  | |
| 1. Stimulate the students to critically assess literature |  | |  | |  | |  | |  | |
| 1. Encourage personal interest and enthusiasm for research in a given field |  | |  | |  | |  | |  | |
| 1. Have sufficient time to support students in the learning process |  | |  | |  | |  | |  | |
| 1. Explain the subject matter deeply, effectively, and clearly |  | |  | |  | |  | |  | |
| 1. Explain the difficult topics smoothly |  | |  | |  | |  | |  | |
| 1. Encourage students to ask critical questions |  | |  | |  | |  | |  | |
| **Perception of students about integration of research in the courses** | | | | | | | | | | |
| 1. My learning is stimulated when education is grounded in research | |  | |  | |  | |  | |  |
| 1. It is important to me that my instructors conduct research | |  | |  | |  | |  | |  |
| 1. Education in which scientific research is central stimulates my learning | |  | |  | |  | |  | |  |
| 1. My understanding of the most important concepts in the domain increases when research is more valued | |  | |  | |  | |  | |  |
| 1. I am enthusiastic about my scientific domain when knowledge is coupled with research | |  | |  | |  | |  | |  |
| 1. Research activity is generally valued in my institution | |  | |  | |  | |  | |  |
| 1. I am more familiar with up-to-date information when research is integrated into education | |  | |  | |  | |  | |  |
| 1. My interest in a topic grows when research is integrated into education | |  | |  | |  | |  | |  |
| 1. My awareness of current research issues is increased when it research integrated into education | |  | |  | |  | |  | |  |
| 1. The research culture in the institution stimulates my learning process | |  | |  | |  | |  | |  |
| 1. I feel more interested in an academic career when research is integrated into education | |  | |  | |  | |  | |  |
| 1. There are opportunities to talk with researchers about scientific research when research is integrated into education | |  | |  | |  | |  | |  |
| 1. The scientific research process is an essential part of the curriculum | |  | |  | |  | |  | |  |
| 1. I feel I am a part of the institution’s academic community when research is integrated into education | |  | |  | |  | |  | |  |
| 1. I am in contact with my instructors’ research when research is integrated into education | |  | |  | |  | |  | |  |
| 1. I want to contribute to the development in my field | |  | |  | |  | |  | |  |
| 1. As a student, I feel involved in research when it is integrated into education | |  | |  | |  | |  | |  |
| 1. I have the opportunity to socially interact with researchers within the institution when research is integrated into education | |  | |  | |  | |  | |  |
| 1. Links to current research practices are made when research is integrated into education | |  | |  | |  | |  | |  |
| 1. I am involved in my instructors’ research when applicable | |  | |  | |  | |  | |  |
| 1. I develop an accurate picture of what is expected of me when research is integrated into education | |  | |  | |  | |  | |  |

| **Student evaluation of teaching quality (SET37) [2]** | | | | | |
| --- | --- | --- | --- | --- | --- |
|  | **Strongly disagree** | **Disagree** | **Neutral** | **Agree** | **Strongly agree** |
| **Think about an instructor of yours with the highest research activity and answer the following questions by applying it to their specific course(s) and teaching methods:** | | | | | |
| 1. The instructor clearly specified what should be learnt and accomplished at the end of the course |  |  |  |  |  |
| 1. The various themes were well geared to one another |  |  |  |  |  |
| 1. The instructor explained the subject matter well and was knowledgeable of the topic |  |  |  |  |  |
| 1. The study materials were useful |  |  |  |  |  |
| 1. The expectations of the instructor of what should have been learned at the end of a course were realistic and acceptable |  |  |  |  |  |
| 1. The instructor helped students with questions and problems that arose during the course |  |  |  |  |  |
| 1. Overall, I was satisfied with the course |  |  |  |  |  |
| 1. I feel that I have learned a lot during the course |  |  |  |  |  |
| 1. I feel that courses are relevant to my educational program |  |  |  |  |  |
| **Think about an instructor of yours with the lowest research activity and answer the following questions by applying it to their specific course(s) and teaching methods:** | | | | | |
| 1. The instructor clearly specified what should be learnt and accomplished at the end of the course |  |  |  |  |  |
| 1. The various themes were well geared to one another |  |  |  |  |  |
| 1. The instructor explained the subject matter well and was knowledgeable of the topic |  |  |  |  |  |
| 1. The study materials were useful |  |  |  |  |  |
| 1. The expectations of the instructor of what should have been learned at the end of a course were realistic and acceptable |  |  |  |  |  |
| 1. The instructor helped students with questions and problems that arose during the course |  |  |  |  |  |
| 1. Overall, I was satisfied with the course |  |  |  |  |  |
| 1. I feel that I have learned a lot during the course |  |  |  |  |  |
| 1. I feel that courses are relevant to my educational program |  |  |  |  |  |

| **Adapted-Teachers’ quality assessment questionnaire (A-TQAQ) [3]** | | | | | |
| --- | --- | --- | --- | --- | --- |
|  | **Most likely** | **Likely** | **Neutral** | **Unlikely** | **Most unlikely** |
| **Think about the qualifications (higher degrees and more advanced diplomas) of instructors in your institution to answer the following statements:** | | | | | |
| **Academic Qualifications** | | | | | |
| 1. All instructors have academic qualifications to teach the students at a senior level |  |  |  |  |  |
| 1. Instructors' academic qualifications influence the students' academic achievements |  |  |  |  |  |
| 1. Instructors' academic qualifications are determinants of their quality |  |  |  |  |  |
| 1. Instructors with higher degrees are more effective in the classroom |  |  |  |  |  |
| 1. Excellent mastering of one’s subject as an instructor is dependent on one's academic qualification |  |  |  |  |  |
| 1. The research activity of an instructor is dependent on one’s academic qualification |  |  |  |  |  |
| 1. Excellent mastering of one’s subject as an instructor is dependent on one’s research activity |  |  |  |  |  |
| 1. Students taught by more experienced researchers perform better academically |  |  |  |  |  |
| 1. A researcher is a role model for students |  |  |  |  |  |
| **Professional Qualifications** |  |  |  |  |  |
| 1. The majority of instructors in this school have professional teaching and instructors' certifications |  |  |  |  |  |
| 1. Instructors with professional teaching qualification(s) have better teaching skills to impart knowledge to students |  |  |  |  |  |
| 1. Instructors with professional teaching qualification(s) have better students' assessment and evaluation skills |  |  |  |  |  |
| 1. Instructors with professional teaching qualification(s) keep better records of students and their performances |  |  |  |  |  |
| 1. The quality of instructors in the school will affect students' academic achievement |  |  |  |  |  |
| **Teaching Experiences** |  |  |  |  |  |
| 1. The majority of instructors in this school have at least 5 years of teaching experience |  |  |  |  |  |
| 1. Instructors with at least 5 years of experience do better in disseminating knowledge to their students |  |  |  |  |  |
| 1. Instructors with more than 5 years of teaching experience have better knowledge and ability for students' control and class management |  |  |  |  |  |
| 1. Students taught by more experienced instructors perform better academically |  |  |  |  |  |

| **Knowledge questionnaire** |
| --- |
| 1. How would you define a scientific hypothesis?    1. A proposed idea or thought    2. An answer or solution to a question    3. An answer or solution to a question that has a capacity of verification or empirical demonstration    4. A logical deduction of the premises that may or may not be verified empirically |
| 1. How would you define a scientific theory? 2. A speculation or assumption with no or insufficient evidence 3. Scientific hypotheses that can be proven but lack evidence for verification 4. A set of scientific knowledge on a given topic or area 5. A set of hypotheses logically connected to one another, with common background, some of which have been verified |
| 1. How would you define the scientific truth? 2. The truth that will be reached through scientific research 3. The absolute truth 4. A consensus of competent experts 5. A fact that can be found in textbooks 6. Facts that your professors teach you |
| 1. The essential characteristic of science is: 2. All scientific conclusions are temporary 3. A scientific theory cannot merely explain natural phenomena but must somehow also exert influence upon them 4. Rather obvious scientific conclusion does not have to be testable 5. An experiment is not an objective model of nature but serves as an introduction into real research of natural phenomena |
| 1. A scale from 1 to 5 (like grades on an examination) is called: 2. Ratio scale 3. Nominal 4. Ordinal 5. Interval 6. It is not a scale |
| 1. Representativeness is a key characteristic of a: 2. Scientific paper 3. Professional paper 4. Scientific research 5. Sample 6. Population |
| 1. MEDLINE is: 2. The first and best known "online" medical journal 3. The international association of medical informaticians 4. The printed form of the Excerpta Medica 5. An abbreviation (acronym) that lists the parts of the research article 6. A medical database |
| 1. In the previous year, you have published a paper in a prestigious journal. Now you want to check the number of citations your paper has received. The best way to do it would be to search the: 2. Author index of the MEDLINE database 3. Corporate index of the Science Citation Index database 4. Author index of the Current Contents database 5. Citation index of the Science Citation Index database 6. Author index of the Science Citation Index database |
| 1. The part of a scientific paper is: 2. Author's curriculum vitae 3. Letter to the editor enclosed with the paper 4. Description of the timeline 5. Acknowledgment to persons who assisted you during the research |
| 1. All listed rules apply to the process of writing an introduction section of a scientific paper EXCEPT: 2. clearly state why the research has been started 3. do not explain textbook facts 4. do not explain words from the title of the paper 5. make it longer rather than shorter 6. clearly define the question to which your research aims to provide an answer |

| **Attitudes Towards Scientific Research** | | |
| --- | --- | --- |
|  | **No**  **(Unfavorable  attitude)** | **Yes**  **(Favorable  attitude)** |
| 1. Do you feel confident in interpreting and writing a research paper? |  |  |
| 1. Have you ever participated in a research project (apart from mandatory academic projects)? |  |  |
| 1. Have you ever written a scientific paper? |  |  |
| 1. Do you think undergraduate students should participate in research? |  |  |
| 1. Do you think undergraduate students can plan and conduct a research project and write a scientific paper? |  |  |
| 1. Research is of utmost importance for science improvement |  |  |
| 1. I love to be involved in research in my future career |  |  |

**References**

1. Visser-Wijnveen GJ, van der Rijst RM, van Driel JH: **A questionnaire to capture students’ perceptions of research integration in their courses**. *Higher Education* 2016, **71**:471-488.

2. Spooren P, Mortelmans D, Christiaens W: **Assessing the validity and reliability of a quick scan for student’s evaluation of teaching. Results from confirmatory factor analysis and G Theory**. *Studies in Educational Evaluation* 2014.

3. Olawoyin AM, Isuku EJ: **Students’ academic achivement as influenced by teachers’ quality: evidence from Southwest, Nigeria**. *European Journal of Education Studies* 2019, **6**.
