# Supplementary material for: The effect of research on the perceived quality of teaching: a cross-sectional study among university students in Lebanon
Source: BMC Med Educ. 2023 Jan 17;23:31. doi: 10.1186/s12909-023-03998-8 (PMC9843099; doi:10.1186/s12909-023-03998-8)
Supplement: Supplementary file 2 — Additional file 2: TableS1. Factor analysis of the Student Perception of Research IntegrationQuestionnaire (SPRIQ). Table S2. Factor analysis of the Adapted-Teachers’Quality Assessment Questionnaire (A-TQAQ) Questionnaire. Table S3. Factoranalysis of the Student Evaluation of Teaching-short form (SET37-QS). Table S4. Confirmatory factor analysis. [file 12909_2023_3998_MOESM2_ESM.docx]

**Supplementary table**

| **Table S1: Factor analysis of the Student Perception of Research Integration Questionnaire (SPRIQ).** | | | |
| --- | --- | --- | --- |
| **Promax rotated matrix** | | | |
| **Factor** | **Factor 1** | **Factor 2** | **Communalities** |
| The scientific research process is an essential part of the curriculum | 0.88 |  | 0.67 |
| I am in contact with my instructors’ research when research is integrated into education | 0.86 |  | 0.59 |
| I want to contribute to the development of my field | 0.85 |  | 0.66 |
| As a student, I feel involved in research when it is integrated into education | 0.82 |  | 0.69 |
| There are opportunities to talk with researchers about scientific research when research is integrated into education | 0.82 |  | 0.72 |
| I am involved in my instructors’ research when applicable | 0.81 |  | 0.53 |
| I feel I am a part of the institution’s academic community when research is integrated into education | 0.80 |  | 0.68 |
| I have the opportunity to socially interact with researchers within the institution when research is integrated into education | 0.79 |  | 0.65 |
| I develop an accurate picture of what is expected of me when research is integrated into education | 0.78 |  | 0.63 |
| Links to current research practices are made when research is integrated into education | 0.76 |  | 0.67 |
| My awareness of current research issues is increased when research integrated into education | 0.71 |  | 0.65 |
| My interest in a topic grows when research is integrated into education | 0.66 |  | 0.66 |
| Research activity is generally valued in my institution | 0.65 |  | 0.55 |
| The research culture in the institution stimulates my learning process | 0.64 |  | 0.62 |
| I am more familiar with up-to-date information when research is integrated into education | 0.63 |  | 0.59 |
| I am enthusiastic about my scientific domain when knowledge is coupled with research | 0.63 |  | 0.66 |
| Education in which scientific research is central stimulates my learning | 0.61 |  | 0.66 |
| I feel more interested in an academic career when research is integrated into education | 0.60 |  | 0.64 |
| It is important to me that my instructors conduct research | 0.56 |  | 0.59 |
| My understanding of the most important concepts in the domain increases when research is more valued | 0.53 |  | 0.63 |
| My learning is stimulated when education is grounded in research | 0.53 |  | 0.59 |
| Explain the difficult topics smoothly |  | 0.94 | 0.74 |
| Encourage students to ask critical questions |  | 0.90 | 0.67 |
| Explain the subject matter deeply, effectively, and clearly |  | 0.84 | 0.73 |
| Integrate knowledge about research findings |  | 0.81 | 0.64 |
| Encourage personal interest and enthusiasm for research in a given field |  | 0.80 | 0.66 |
| Teach the students to pay attention to recent developments in the field |  | 0.80 | 0.72 |
| Inspire the students to learn more about a given discipline |  | 0.79 | 0.64 |
| Have sufficient time to support students in the learning process |  | 0.78 | 0.66 |
| Teach the students to pay attention to the way research is carried out |  | 0.70 | 0.68 |
| Familiarize the students with the research carried out by the instructors |  | 0.69 | 0.65 |
| Encourage the students not to be satisfied with an explanation too quickly |  | 0.68 | 0.58 |
| Teach the students to pay attention to the research methodology |  | 0.66 | 0.59 |
| Give information about studies that have been carried out in my field |  | 0.63 | 0.60 |
| Encourage the students to search for answers to the unanswered research question |  | 0.44 | 0.49 |
| Stimulate the students to critically assess the literature |  | 0.40 | 0.49 |
| **Percentage variance explained = 63.44%** | 58.40 | 5.04 |  |
| **Cronbach alpha= 0.98** | 0.97 | 0.96 |  |
| **Kaiser-Meyer-Olkin (KMO)= 0.98** |  | | |
| **Bartlett’s test of sphericity p<0.001** |  |  |  |
| Factor 1: Perception of students about research integration;  Factor 2: Beliefs of students towards an educator who is also a researcher. | | | |

| **Table S2: Factor analysis of the Adapted-Teachers’ Quality Assessment Questionnaire (A-TQAQ) Questionnaire.** | | | |
| --- | --- | --- | --- |
| **Promax rotated matrix** | | | |
| **Factor** | **Factor 1**  **Academic qualifications** | **Factor 2**  **Professional qualifications** | **Communalities** |
| The research activity of an instructor is dependent on one’s academic qualification | 0.84 |  | 0.64 |
| Excellent mastering of one’s subject as an instructor is dependent on one's academic qualification | 0.81 |  | 0.63 |
| Instructors with higher degrees are more effective in the classroom | 0.80 |  | 0.56 |
| Instructors' academic qualifications are determinants of their quality | 0.75 |  | 0.61 |
| Excellent mastering of one’s subject as an instructor is dependent on one’s research activity | 0.74 |  | 0.59 |
| All instructors have academic qualifications to teach the students at a senior level | 0.72 |  | 0.47 |
| A researcher is a role model for students | 0.71 |  | 0.63 |
| Instructors' academic qualifications influence the students' academic achievements | 0.63 |  | 0.49 |
| Students taught by more experienced researchers perform better academically | 0.62 |  | 0.57 |
| Instructors with more than 5 years of teaching experience have better knowledge and ability for students control and class management |  | 0.83 | 0.62 |
| Students taught by more experienced instructors perform better academically |  | 0.82 | 0.59 |
| Instructors with a professional teaching qualification(s) have better students’ assessment and evaluation skills |  | 0.76 | 0.59 |
| The majority of instructors in this school have at least 5 years of teaching experience |  | 0.76 | 0.53 |
| Instructors with a professional teaching qualification(s) have better teaching skills to impart knowledge to students |  | 0.73 | 0.63 |
| Instructors with at least 5 years of experience do better in disseminating knowledge to their students |  | 0.72 | 0.54 |
| Instructors with a professional teaching qualification(s) keep better records of students and their performances |  | 0.70 | 0.54 |
| The quality of instructors in the school will affect students' academic achievement |  | 0.68 | 0.54 |
| The majority of instructors in this school have professional teaching and instructors’certifications |  | 0.57 | 0.50 |
| **Percentage variance explained = 57.00** | 48.49 | 8.51 |  |
| **Cronbach alpha= 0.94** | 0.91 | 0.90 |  |
| **Kaiser-Meyer-Olkin (KMO)= 0.93** |  | | |
| **Bartlett’s test of sphericity p<0.001** |  |  |  |

| **Table S3: Factor analysis of the Student Evaluation of Teaching-short form (SET37-QS).** | | | |
| --- | --- | --- | --- |
| **Table 3A: Thinking about the course of an instructor with the highest research activity** | | | |
| **Promax rotated matrix** | | | |
| **Factor** | **Items** | **Factor 1** | **Communalities** |
| The instructor explained the subject matter well and was knowledgeable about the topic | 3 | 0.91 | 0.83 |
| Overall, I was satisfied with the course | 7 | 0.91 | 0.82 |
| I feel that courses are relevant to my educational program | 9 | 0.91 | 0.82 |
| I feel that I have learned a lot during the course | 8 | 0.89 | 0.80 |
| The various themes were well geared to one another | 2 | 0.89 | 0.80 |
| The study materials were useful | 4 | 0.89 | 0.79 |
| The expectations of the instructor of what should have been learned at the end of a course were realistic and acceptable | 5 | 0.89 | 0.79 |
| The instructor helped students with questions and problems that arose during the course | 6 | 0.89 | 0.78 |
| The instructor specified what should be learned and accomplished at the end of the course | 1 | 0.87 | 0.76 |
| **Percentage variance explained** | 79.79 | | |
| **Cronbach alpha** | 0.97 | | |
| **Kaiser-Meyer-Olkin (KMO)** | 0.96 | | |
| **Bartlett’s test of sphericity** | <0.001 | | |
|  | | | |
| **Table 3B: Thinking about the course of an instructor with the lowest research activity** | | | |
| **Promax rotated matrix** | | | |
| **Factor** | **Items** | **Factor 1** | **Communalities** |
| Overall, I was satisfied with the course | 7 | 0.88 | 0.77 |
| The instructor explained the subject matter well and was knowledgeable about the topic | 3 | 0.87 | 0.75 |
| I feel that I have learned a lot during the course | 8 | 0.86 | 0.75 |
| The expectations of the instructor of what should have been learned at the end of a course were realistic and acceptable | 5 | 0.86 | 0.73 |
| I feel that courses are relevant to my educational program | 9 | 0.84 | 0.71 |
| The study materials were useful | 4 | 0.84 | 0.71 |
| The instructor helped students with questions and problems that arose during the course | 6 | 0.84 | 0.70 |
| The various themes were well geared to one another | 2 | 0.82 | 0.67 |
| The instructor specified what should be learned and accomplished at the end of the course | 1 | 0.81 | 0.65 |
| **Percentage variance explained** | 71.48 | | |
| **Cronbach alpha** | 0.95 | | |
| **Kaiser-Meyer-Olkin (KMO)** | 0.95 | | |
| **Bartlett’s test of sphericity** | <0.001 | | |

| **Table S4: Confirmatory factor analysis** | | | | |
| --- | --- | --- | --- | --- |
|  | SPRIQ | Adapted-Teachers’ Quality Assessment Questionnaire (A-TQAQ) | Student Evaluation of Teaching-short form (highest research activity) | Student Evaluation of Teaching-short form (lowest research activity) |
| Χ^2^/df | 3.08 | 4.73 | 4.15 | 3.84 |
| GFI | 0.794 | 0.862 | 0.942 | 0.953 |
| AGFI | 0.769 | 0.824 | 0.906 | 0.922 |
| CFI | 0.911 | 0.889 | 0.980 | 0.977 |
| TLI | 0.906 | 0.874 | 0.975 | 0.969 |
| RMSEA | 0.069  (0.065; 0.072) | 0.092  (0.085; 0.099) | 0.084  (0.069; 0.100) | 0.080  (0.064; 0.097) |
| Χ2/df : Relative Chi-square  GFI: Goodness of Fit Index  AGFI: Adjusted Goodness of Fit Index  CFI: comparative fit Index  TLI: Tucker Lewis Index  RMSEA: Root Mean Square Error of Approximation  SPRIQ: Student Perception of Research Integration Questionnaire | | | | |
